# Can Decision Biases Improve Insurance Outcomes? An Experiment on *Status Quo* Bias in Health Insurance Choice

**DOI:** 10.3390/ijerph10062560

**Published:** 2013-06-19

**Authors:** Miriam Krieger, Stefan Felder

**Affiliations:** 1 Fakultät für Wirtschaftswissenschaften, Universität Duisburg Essen, Schützenbahn 70, 45127 Essen, Germany; E-Mail: Miriam.Krieger@uni-due.de; 2 Wirtschaftswissenschaftliche Fakultät, Universität Basel, Peter Merian-Weg 6, CH 4002, Basel, Switzerland; 3 CINCH—Health Economics Research Center, Edmund-Körner-Platz 2, D-45127 Essen, Germany

**Keywords:** experimental health economics, health insurance, liberal paternalism

## Abstract

Rather than conforming to the assumption of perfect rationality in neoclassical economic theory, decision behavior has been shown to display a host of systematic biases. Properly understood, these patterns can be instrumentalized to improve outcomes in the public realm. We conducted a laboratory experiment to study whether decisions over health insurance policies are subject to *status quo* bias and, if so, whether experience mitigates this framing effect. Choices in two treatment groups with *status quo* defaults are compared to choices in a neutrally framed control group. A two-step design features sorting of subjects into the groups, allowing us to control for selection effects due to risk preferences. The results confirm the presence of a *status quo* bias in consumer choices over health insurance policies. However, this effect of the default framing does not persist as subjects repeat this decision in later periods of the experiment. Our results have implications for health care policy, for example suggesting that the use of non-binding defaults in health insurance can facilitate the spread of co-insurance policies and thereby help contain health care expenditure.

## 1. Introduction

Under the neo-classical paradigm of economics, individuals are assumed to make rational decisions on the basis of well-defined preferences. However, ample evidence for systematic deviations of decision behavior from this notion has challenged the validity of *homo economicus* as a comprehensive descriptive model [1]. One of the observed anomalies is a preference for the *status quo*. Termed the “*status quo* bias” by Samuelson and Zeckhauser [2], it describes a phenomenon where the characteristics of the initial situation, rather than only those of the available alternatives themselves, influence an individual’s choice. This violates central axioms of rational choice such as invariance and independence of irrelevant alternatives [3]. *Status quo* bias has been observed in a variety of contexts including consumption, savings, travel, organ donations, pharmacy choice, vaccination rates, and experimentally in stock trading and contributions to a public good [4,5,6,7,8,9,10,11,12]. The general finding of this literature is that in decisions containing a *status quo* or default option, choices are significantly influenced by and towards this option. 

Decision biases should be particularly salient in health insurance, where complex choices are made under uncertainty and evaluating all contingencies and probabilities is difficult. From an individual’s perspective, the expected utility-maximizing insurance contract will under most conditions, e.g., risk-aversion and net premium loading [13], feature cost sharing by the insured [14,15]. From a societal point of view, co-insurance is necessary to address moral hazard and contain the demand for health care [16,17]. Nevertheless, individuals tend to over-insure, *i.e.*, to purchase too much insurance coverage [18]. Such preferences for full-coverage policies in car, liability, and health insurance have even been described as “deductible aversion” [19]. 

If *status quo* bias is found to exist in health insurance choice, there are clear implications for policy. In the vein of libertarian paternalism [20,21], *status quo* bias could be harnessed to facilitate welfare-enhancing choices, e.g., by making co-insurance the default option in public health systems. A default position exists for all decisions—it is what happens if no active choice is made. For decisions in the public realm, the public hand thus has an opportunity to foster more rational market outcomes by setting appropriate defaults, *i.e.*, defining which alternative shall come into effect if an individual makes no choice of his own. If this default is non-binding, rational individuals with divergent preferences remain free to choose otherwise without incurring additional costs. In practice the default option often, though not necessarily, coincides with the *status quo*: the current state continues unless an alternative is chosen. 

The *status quo* bias is usually explained by reference to non-rational behavior such as cognitive misperceptions and psychological or emotional biases [2,22]. These include loss aversion [23], anchoring and incomplete adjustment [24], sunk cost fallacy [25], regret aversion [26], and avoiding cognitive dissonance [2], to name but a few.

Several previous studies investigate *status quo* bias in insurance decisions in general. In early research on the demand for car liability insurance, comparing survey and field data for groups with different *status quo* settings, most individuals were found to retain their *status quo* option [27]. Two studies using field data on health plan selection by university employees find that choices differ significantly between new and old enrollees, indicating *status quo* inertia [2,28]. A survey of insurance choices in Switzerland also suggests evidence for *status quo* bias, as individuals with longer plan tenure express less desire to switch [29]. 

*Status quo* bias has also been addressed in health insurance decisions in two studies. However, the research design employed in both is not primarily intended to test for this effect, which makes it difficult to interpret the results in these terms. Schram and Sonnemans analyzed the determinants of health insurance choice by simulating the Dutch health insurance market in a laboratory experiment [30]. They find subjects to switch health plans more often than is rational—despite switching costs—and interpret this as evidence for the lack of *status quo* bias. However, their experiment discerns switching between plans, but not relative to a reference point such as a *status quo* or default. Frequent switching in later periods could be due to experience (we discuss this below). Similarly, Zweifel *et al.* consider the lack of switching between policies as indication for *status quo* bias in health insurance decisions [31]. They conducted discrete choice experiments in Germany and The Netherlands to establish the willingness-to-pay for health insurance attributes and find a substantial willingness-to-pay among their subjects to retain the current policy. But this also need not imply non-rational behavior, e.g., if the current policy is utility-maximizing. Hence, neither study is focused strictly enough on the *status quo* bias to provide a true test for this effect in health insurance decisions.

Our research adds to this literature both methodologically and by contributing to the understanding of health insurance choices. Firstly, we use an economic laboratory experiment to specifically investigate the occurrence of *status quo* bias in health insurance choice. Nearly all previous studies are based on the analysis of field data, natural experiments, and surveys. While Altmann and Falk [12] do use an incentivized experiment to test for *status quo* bias—for which they find evidence—their results from a public goods game do not necessarily extend to insurance decisions. Schram and Sonnemans [30] is also a fully incentivized laboratory experiment, though, the focus of its experimental design lies elsewhere and does not precisely address the *status quo* bias. 

Laboratory experiments have become an important means of collecting data in economics and other social sciences [32,33]. They permit the study of behavior within tightly regulated environments under true ceteris paribus conditions. In contrast to research based on field data, decision elements that might confound the *status quo* effect, such as search and information costs or an excess number of alternatives, can be controlled for in the laboratory. In particular, our experimental design controls for the influence of risk preferences on insurance choice by featuring sorting of the subjects into the treatment groups. If *status quo* bias can be identified under these conditions in the laboratory, it is even more likely to prevail in a more natural setting.

Accordingly, the first research question we seek to answer is:

R1: Are choices over health insurance policy in a laboratory experiment subject to *status quo* bias?

Based on the previous research, we expect decision behavior in our treatment groups to deviate from that in the control group in accordance with their *status quo* defaults: subjects initially insured under a full-coverage policy should on average select lower co-insurance levels than the control group; inversely, subjects who start out with a high co-insurance arrangement should select higher co-insurance levels on average. That is, if *status quo* bias holds for decisions over health insurance policies, subjects are expected to make their choices for higher or lower co-insurance based not only on their risk preferences, but also on their decision environment, in this case the *status quo* default policy.

A further issue our research addresses is the effect of experience on the *status quo* bias. Several studies suggest that non-rational decision anomalies fade as individuals gain experience. Löfgren *et al.* conduct an online experiment of the *status quo* bias and find no evidence for such behavior among their expert subjects—environmental economists choosing whether to offset CO_2_-emissions from traveling to a professional conference [34]. Pertaining to health insurance, Shapira and Venezia find that in an experiment with professional insurance brokers and inexperienced MBA-students only the latter underestimated the value of insurance policies featuring deductibles [35]. Their explanation is that the students anchor their choice on the price of the full-coverage policy and fail to adjust fully towards the co-insurance option, accounting for the cheaper premium but not probability-weighting the deductible. In field experiments in markets for collectors’ items List finds that the behavior of inexperienced participants generally fits the predictions of prospect theory, while choices by experienced individuals is better explained by expected utility maximization [36,37]. Similarly, in an experiment involving students and professional traders List and Haigh show that the Allais Paradox is less common among the latter [38]. 

In our experiment, we examine the effect of experience on *status quo* bias by recruiting inexperienced subjects and employing a repeated-choice design that allows them to gain experience in the task at hand over the course of several periods. Therefore, our research question here is

R2: If *status quo* bias is observed initially in a laboratory experiment on health insurance choice, does it persist after the first decision period?

If experience does erode the *status quo* bias, we would expect choices in the treatment and control groups to converge after the first period of the experiment.

Our laboratory experiment studies whether decisions over health insurance policies with different co-insurance levels are subject to *status quo* bias. We compare the behavior of two treatment groups for which decisions were given a *status quo* default to that of a control group facing a neutrally framed choice. A two-step design with risk sorting of subjects into the groups permits the isolation of this framing effect from the influence of risk preference distributions. We also investigate whether gaining experience in the decision ameliorates the framing effect by employing a repeated-choice design. Our main result is that the first decision is significantly characterized by *status quo* bias. This confirms previous research on *status quo* bias in other contexts. In repetitions of the decision the framing effect is no longer observable. This result, too, is consistent with studies suggesting that experience counteracts the *status quo* bias. Our study adds to a growing body of experimental research on health economics topics, answering to calls for incorporating experimental and behavioral approaches into health economics [39,40,41].

## 2. Experimental Design

In order to study *status quo* bias in health insurance decisions we employ a two-step experimental design. The first part is an online experiment aimed at measuring the participants’ risk attitudes. In the second and main part, we study the effect of a *status quo* default in the laboratory by comparing health insurance choices made by two treatment groups against those of a control group. One of the treatment groups has a full coverage insurance policy as *status quo* default, the other a maximum co-insurance policy, and the control group has no decision default at all. 

Since risk preferences are central to explaining insurance decisions—*ceteris paribus*, more risk-averse individuals should choose lower co-insurance levels [14,42,43]—it is important to isolate the effect of this factor from that of our treatment variable, the *status quo* default framing. We therefore clustered our subjects into the treatment and control groups in such a way that the three groups were homogeneous with regards to the distributions of their members’ risk preferences (this is described in more detail below). To our knowledge, this type of risk sorting is a novel feature in economic experiments.

Subjects were informed in the invitation that the experiment would take place in two parts, one online and one in the laboratory. They were also told before signing up for the experiment that they could only receive a payoff if they participate in both parts. 

### 2.1. Online Test for Risk Preferences

**Figure 1:**
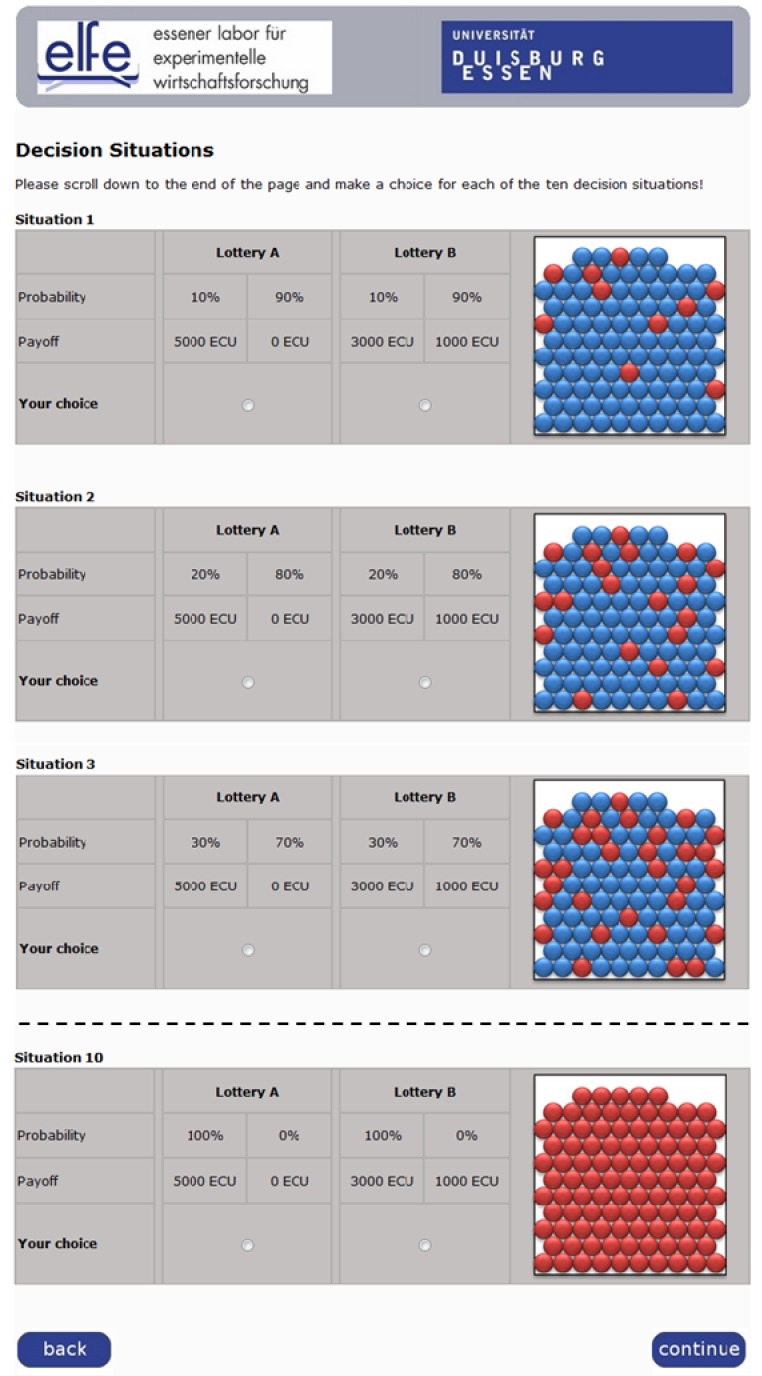
Screenshot (extract) of the online test for risk preferences.

We used an online experiment to determine subjects’ risk preferences by means of a multiple price choice task similar to that introduced by Holt and Laury [44,45], perhaps the most common experimental method for risk preference elicitation [46]. Our subjects faced ten situations in which they had the choice of a pair of lotteries, one always riskier and one safer. As Figure 1 shows, all decisions appeared on the same screen and probabilities were illustrated using urns with colored balls. 

In the first situation, the riskier lottery yields 5,000 ECU (“experimental currency unit”, 600 ECU = 1 Euro; the same exchange rate was used throughout the experiment to keep the stakes in all decisions aligned) with a probability of 0.1 and 0 ECU with a probability of 0.9 and the safer lottery yields 3,000 ECU with a probability of 0.1 and 1,000 ECU with a probability of 0.9. In the subsequent decisions, probabilities in both lotteries increase in increments of 0.1 while the possible payoffs remain fixed. In the final decision, the payoff of 5,000 ECU (3,000 ECU) carries a probability of 1.0 and the payoff of 0 ECU (1,000 ECU) one of 0. Only very risk-loving subjects would choose the risky lottery in the first decision. All others would choose the safer option and switch over to the riskier lottery in the course of the ten decisions. This switching point is a proxy for the degree of risk aversion: the later the switch, or the more safe choices made, the more risk-averse the subject. A risk neutral individual would select the safer lottery three times and the riskier lottery seven times in this experiment. All subjects should have switched to the riskier choice by the last situation, preferring the certain 5,000 ECU to the certain 3,000 ECU. 

The lottery decisions in the online experiment were incentivized by the random payment technique, where one of the ten decision situations is drawn at random and the lottery chosen in this situation realized to establish the payoff. This procedure is widely used in economic experiments to avoid averaging and income effects in repeated decisions. (Random payment also has other advantages, such as maximizing the yield of observations for a given research budget. While concerns have been raised as to whether it dilutes the power of the monetary incentive, research on this question generally reports no adverse effect of random payment for non-complex choice tasks [47,48,49]. In an experiment that specifically analyzes this problem no significant differences are found between behavior in treatments where all ten decisions are paid off and treatments where one of the same ten decisions is paid off, although increasing the scale of payoffs overall does influence choice behavior [50]). In order to avoid biasing later decisions through income effects, subjects did not learn of or receive their payoff for the online part until the laboratory part was completed as well.

Based on the number of safe choices subjects made in the online experiment, we organized our subjects into three homogeneous groups for the laboratory section of the experiment. Subjects were allocated to these groups randomly by an algorithm which minimized the differences in mean and variance of the number of safe choices between each pair of groups. 

### 2.2.Laboratory Experiment to Test for Status Quo Bias

The laboratory section comprised an individual decision task in a repeated design with four periods. In all periods, subjects had the choice of five health insurance policies (options A through E) with different co-insurance arrangements, requiring respective copayments of 0%, 20%, 30%, 40%, and 50% of any medical expenses incurred. 

#### 2.2.1. Sequence of Events in Each Period

**Figure 2:**
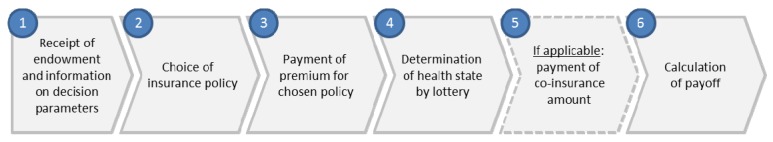
Sequence of steps in each period of the laboratory experiment.

Figure 2 illustrates the basic sequence of events, which was repeated identically in each period. In the first step of each period, every subject received an endowment of 2,000 ECU as well as information on the probability with which he would fall ill during this period and the cost of the medical treatment he would require in this case. In step two, subjects chose a health insurance policy from the five available options. In the third step, a stochastic process determined whether the subject fell ill or not, based on the probability of illness for that period. This random draw was carried out once per period for all subjects, thus holding health status in each period constant across all subjects. If he did contract a disease, the subject was told that he received medical treatment which fully restored his health. Subjects were instructed that health states in the four periods were independent of each other. Depending on which policy he chose, the subject might have to pay for part of the incurred medical expenses using his remaining endowment. The balance of the endowment—net of the premium and the coinsurance amount—made up the subject’s payoff for this period. Providing subjects with an endowment which he uses to buy insurance and medical care if needed reflects the situation outside the laboratory in which individuals use their existing wealth to cover such expenses; in both cases, purchase decisions are made in the domain of losses (according to prospect theory, people fear a loss more than they value a gain of equal size; see [51]).

#### 2.2.2. Default Framing as Treatment Variable

The main treatment variable in our experimental design is the framing of the decision situation. This allows us to address our primary research question on whether *status quo* bias bears on health insurance decisions. The first treatment group FULL was given a full coverage insurance policy (option A, 0% co-insurance) as *status quo* default for the decision. The second treatment group SHARE had the maximum co-payment policy (option E, 50% co-insurance) as *status quo* default. The BASELINE control group had no *status quo* or default setting at all and was hence forced to take “active decisions” [5]. 

**Figure 3:**
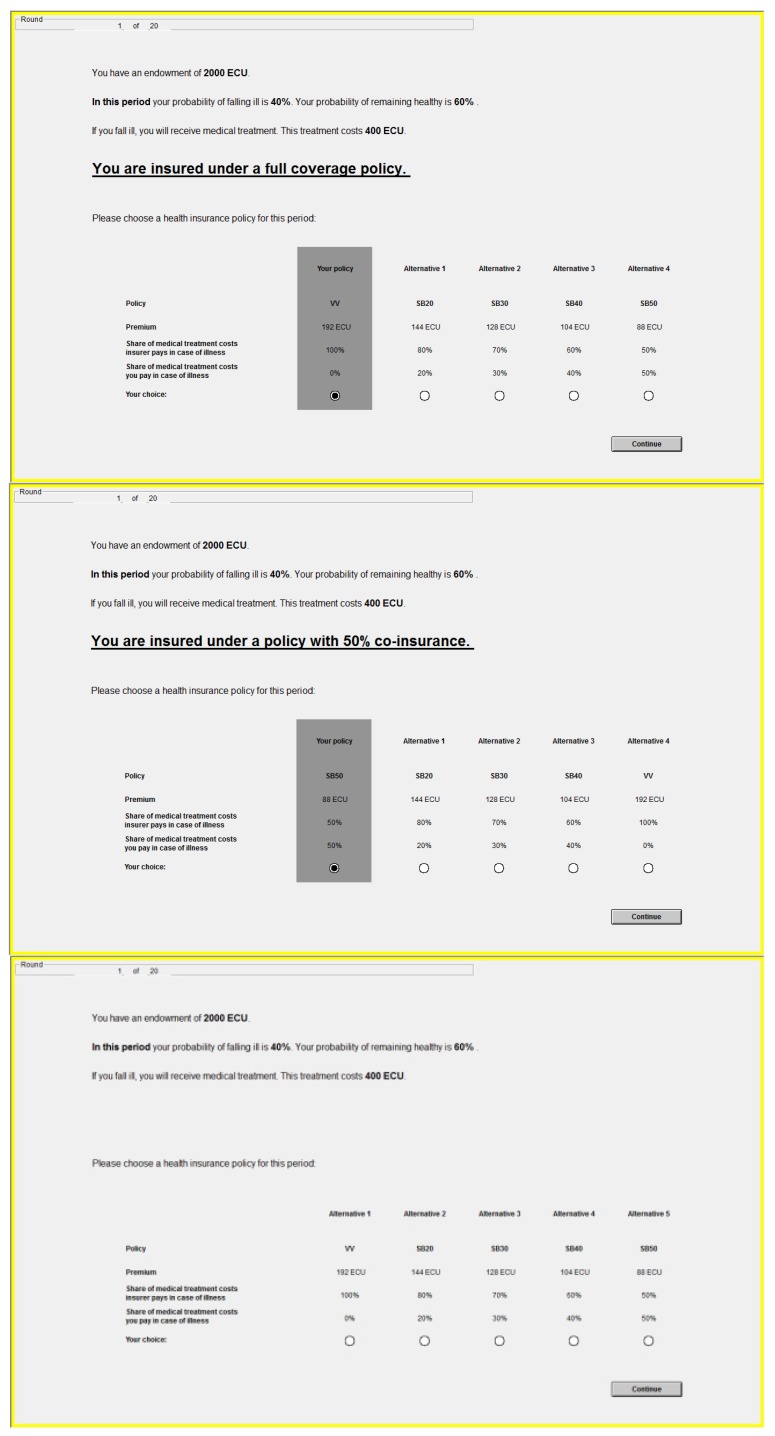
The decision situation for groups FULL, SHARE, BASELINE (top to bottom).

This default *status quo* framing was embedded in the experimental decision environment in two ways. Firstly, the instructions provided to the subjects in groups FULL and SHARE at the beginning of the laboratory part of the experiment clearly stated and described their current health insurance policies, *i.e.*, the *status quo*. Secondly, these default policies were restated again on the computer screen in every decision situation and emphasized visually by an underlying box and a pre-selected radio button (see Figure 3). However, these policy defaults were non-binding: subjects were free to diverge from this option at the cost of a mouse click. Subjects in the control group BASELINE received instructions which did not mention a current or *status quo* insurance situation (but were otherwise identical to those for the treatment groups); and their decision screens did not contain the visual cues indicating a decision default.

#### 2.2.3. Experience through Repeated Design

As our second research question deals with the effect of experience on *status quo* bias, we gave the subjects the opportunity to gain experience in choosing health insurance policies by repeating the task over four periods. The framing in the treatment and control groups as well as the co-insurance levels of the five policy options were held constant across all periods, while other decision parameters varied (see next section). Note that the decisions in the four periods are independent of each other; they represent different potential health situations the subject might face rather than a temporal sequence of connected decisions. Subjects therefore did not learn the outcome of each decision until the entire experiment was completed. This way knowledge of their health outcome (and the consequent medical expenses) in one period could not bias insurance choices in subsequent periods. Thus, in step 4 of all periods subjects were told that the computer was now stochastically determining their health state and shown what the consequences of both possible states would be for their payoff. But they did not see the result of this lottery until the end of the fourth period. Comparing the differences between the treatment groups and the control group in period 1 to the differences in the following periods provides information on the effect of task repetition, or experience, on *status quo* bias. Our results indicate that this number of repetitions, while low, appears to be sufficient to make a change in behavior observable.

#### 2.2.4. Decision Parameters

In every period, all subjects faced the same decision parameters. The probability of falling ill as well as the cost of medical treatment in the event of illness varied across the four periods (see Table 1). 

**Table 1:** Decision parameters available to the subjects.

Period	Probability of illness	Cost of treatment (ECU)	Premium (ECU) Risk premium (ECU) Expected value (ECU)				
			Policy A (0% co-insurance)	Policy B (20%)	Policy C (30%)	Policy D (40%)	Policy E (50%)
Period 1	0.40	400	192.0	144.0	128.0	104.0	88.0
			32.0	16.0	16.0	8.0	8.0
			1,808.0	1,824.0	1,824.0	1,832.0	1,832.0
Period 2	0.20	800	200.0	160.0	136.0	112.0	88.0
			40.0	32.0	23.0	16.0	8.0
			1,800.0	1,808.0	1,816.0	1,824.0	1,832.0
Period 3	0.10	1,500	187.5	150.0	127.5	105.0	82.5
			37.5	30.0	22.5	15.0	7.5
			1,812.5	1,820.0	1,827.5	1,835.0	1,842.5
Period 4	0.03	3,000	112.5	90.0	76.5	63.0	49.5
			22.5	18.0	13.5	9.0	4.5
			1,887.5	1,892.0	1,896.5	1,901.0	1,905.5

The premiums for the five policies varied as well, as they were calculated as an actuarially fair premium (insurer’s share of the cost of treatment weighted by the probability of illness) plus a loading factor representing the insurer’s transaction costs. The loading factor, or risk premium, is higher the smaller the co-insurance requirement. This serves to offset somewhat the effect of risk-aversion, which favors options with less variance. Furthermore, including a loading factor mirrors common practice outside the laboratory where it is typically incorporated into insurance contracts e.g., as an incentive to foster health prevention by the insured, or as reflecting lower costs of claims processing if co-insurance reduces the demand for treatment [15].

#### 2.2.5. Determining Subject Payoff

The laboratory section of the experiment was also incentivized by random payment of one decision. The period drawn at random was then realized five times using the choice made by the subject. Steps 1-6 as described above were first carried out once, and then repeated four more times while skipping step 2. The sum of the payoffs in these five realizations made up the subjects’ total payoff from the laboratory task. This looped procedure served to increase the feedback subjects received on their choices with the aim of inducing more deliberate decisions. It reflects a situation outside the laboratory in which individuals can fall ill (and require treatment, with the pursuant financial consequences) between zero and several times within the period for which they pay a health insurance premium, e.g., a month or a year.

### 2.3. Experimental Protocol

Seventy (70) subjects participated in both the online and the laboratory sections of the experiment (see Table 2). A further 19 subjects participated in the online part only. Among these, six were not admitted to the laboratory due to incomplete or inconsistent online results (we discuss this below in the results section) and 13 subjects did not appear for the laboratory part (forfeiting their earnings from the online task). The subjects were recruited using ORSEE [52] among students who enrolled at Duisburg-Essen University prior to 2007. Using a student subject pool is helpful in addressing our research question R2 on experience and the *status quo* bias, as they are very unlikely to have any prior experience in choosing health insurance. University students in Germany are generally insured under standard student policies and do not make substantial (or often any) insurance-related choices until they enter the labor market. In order to maintain an inexperienced subject pool we also excluded students of economics, business administration, engineering economics and business informatics, who are expected to have encountered topics related to (health) insurance in the course of their studies. 

**Table 2:** Participants in the experiment.

	Session 1	Session 2	Session 3	Total
Participated online	32	29	28	89
Admitted to laboratory	29	27	27	83
Participated in laboratory	24	24	22	70

After signing up for the experiment, subjects received an e-mail with the URL and a four digit access code for the online part of the experiment. (This code was also used to anonymously match the data from the online and the laboratory sections.) Subjects received detailed instructions at the beginning of the online experiment which described the subsequent task and how their payoff would be determined. Completing the online experiment took 10–20 min. 

The laboratory part of the experiment took place in three 90 min sessions in June and July 2010 at the Essener Labor für Experimentelle Wirtschaftsforschung (elfe) laboratory. The experiment was carried out using the specialized software z-Tree [53].

Subjects received their payoff for both parts of the experiment after completing the laboratory section. Those who participated in the entire experiment earned an average of € 24.55 (min. € 22.73, max. € 26.50), which consists of € 6.62 for the online part (min. € 5.00, max. € 8.33), € 14.93 for the laboratory section (min. € 14.73, max € 15.17), and a show-up fee of € 3.00. Subjects who did not participate in the laboratory were also asked to collect their payoff for the online experiment there. They earned an average of € 6.67 (min. €5.00, max. € 8.40) plus a show-up fee of € 5.00, totaling € 11.67 (min. € 10.00, max. € 13.40).

## 3. Results and Discussion

### 3.1. Risk Preferences

In the online test for risk aversion subjects made an average of 5.26 safe choices (SD 2.23), thus generally exhibiting a moderate degree of risk aversion (see Table 3). This is well in line with the findings of Holt and Laury, where subjects chose the safe lottery between 5.0 and 5.5 times on average in the treatments with stakes similar to those in this experiment [44].

**Table 3:** Number of safe choices by group.

Group	Mean	Median	SD	N
Full	5.35	5	2.15	23
Baseline	5.22	5	2.22	23
Share	5.21	6	2.34	24
All	5.26	5	2.23	70

As indicated above, six out of 89 subjects were not admitted to the laboratory part of the experiment based on their results in the online test. These subjects either switched between the safer and the riskier lottery more than once or selected the dominated safe option in the final situation. There are various possible explanations for this type of behavior: subjects might simply have not understood the lottery choice task. Or they did not understand the random payment method and made a “portfolio” of choices across the ten decision situations. In theory, individuals could also have different risk preferences even within the narrow range covered in our experiment, though none of our subjects exhibited behavior apparently compatible with this explanation. In any event, this type of inconsistent decision behavior applies to barely 7% of the subjects in the online experiment, a share which is low compared to the 7–13% reported by Holt and Laury for the comparable treatments [44].

The purpose of the risk preference elicitation task was to form homogeneous groups and thus control for the influence of risk preference distributions on the insurance choice. Pairwise comparisons of the groups using a Mann-Whitney U test (exact, two-sided) confirm that there is no statistically significant difference regarding the means and variances of the number of safe choices made in the online task (p ≥ 0.797).

### 3.2. Status Quo Bias

In addressing our research question R1 whether decisions on health insurance policy are subject to *status quo* bias, we first consider only the entirely independent observations from the first period so as to avoid order effects. A descriptive summary of the average levels of co-insurance chosen in each group is presented in Table 4. 

**Table 4:** Level of co-insurance (%) selected in period 1.

Group	Mean	Median	SD	N
Full	23.0	20.0	16.9	23
Baseline	32.1	35.0	16.4	24
Share	33.5	40.0	14.0	23

In the first period, subjects in the BASELINE group selected an average co-insurance of 32.1% (recall that the minimum choice is 0% co-insurance and the maximum is 50%). Subjects in treatment group FULL chose distinctly less co-insurance, 23.0% on average. This difference is significant at the 5%-level using both the Mann-Whitney-U test (p = 0.034) and the more rigorous Kolmogorov-Smirnov-test (p = 0.027; both exact and two-sided) (in our case even a t-test provides very similar results: p ≤ 0.070 for the comparison FULL/BASELINE; p ≤ 0.756 for SHARE/BASELINE; and p = 0.028 for FULL/SHARE). Group SHARE chose slightly more co-insurance than the control group, 33.5%, though the difference is not significant (p ≥ 0.918). The difference in choices between the two treatment groups is of course also statistically significant (p ≤ 0.045). One reason why we detect significant *status quo* bias here in group FULL but not in group SHARE might be that the full coverage option provides a stronger anchor than the 50% co-insurance policy. “Zero” is always a special reference point in a decision situation [24,54]. In fact, the *status quo* bias displayed by group FULL appears to be substantial enough to outweigh the slight skew towards the co-insurance contracts in the expected values of the five options which is caused by loading the insurance premiums as described in Section 2.2.4. On the other hand, this skew in the premium design would obscure an underlying *status quo* bias effect in group SHARE. Outside the highly abstracted laboratory environment, further factors such as switching costs would be expected to add to an individual’s decision inertia and substantiate the effect of the *status quo* bias.

We also estimated an ordered probit model of the co-insurance amount chosen, using variables for the affiliation with treatment groups FULL and SHARE as well as risk preference on the right-hand side (see Table 5). We apply the model separately to each period and to the average of the choices made by each subject in all periods. The results for period 1 confirm the findings from the nonparametric statistical tests: Membership of treatment group FULL is associated with selecting policies with significantly lower levels of co-insurance than membership of the group BASELINE. The coefficient for membership of group SHARE carries the expected sign, but is not statistically significant. Furthermore, risk aversion—indicated by the number of safe choices in the online experiment—is significantly and negatively correlated with the amount of co-insurance chosen (p ≤ 0.1) in periods 1 to 3 (in period 4 the coefficient for risk aversion carries the expected sign but is neither economically nor statistically significant.) This, too, is in line with the results of our nonparametric statistical tests and confirms the consistency of our subjects’ decisions. It also coincides with other empirical research supporting the theoretical finding that higher co-insurance amounts are utility-maximizing for more risk-averse individuals [42,43].

**Table 5:** Ordered probit estimation coefficients for co-insurance levels chosen. ^‡^

Amount of co-insurance chosen	Description	Period 1	Period 2	Period 3	Period 4	All Periods ^a^
Dummy FULL	1 if group = FULL, 0 otherwise	−0.644 * (0.337)	−0.272 (0.337)	−0.034 (0.354)	0.320 (0.351)	−0.194 (0.302)
Dummy SHARE	1 if group = SHARE, 0 otherwise	0.042 (0.309)	0.146 (0.352)	0.209 (0.230)	0.291 (0.298)	0.216 (0.312)
Risk aversion	Number of safe choices, 0 to 10	−0.121 ** (0.058)	−0.095 * (0.058)	−0.103 * (0.054)	−0.072 (0.063)	−0.133 ** (0.056)
No. of obs.		70	70	70	70	70
Log pseudo-likelihood		−104.9	−94.9	−107.3	−100.5	−184.9
Pseudo-R^2^		0.0459	0.0213	0.0178	0.0128	0.0200

^‡^ Estimating the ordered probit without risk aversion yields the same qualitative results; ^a^ Dependent variable: avg. co-insurance per subject across all periods; */** signify significance at 10%/5% level of confidence; Robust standard errors, clustered by subject for all periods.

We also controlled for additional subject characteristics in a more comprehensive specification of the model. In a short post-experimental survey, we gathered information on subjects’ gender, age, insurance status (SHI or private), supplemental insurance ownership, previous experience with health insurance decisions, and familiarity with SHI alternative policies (“GKV Wahltarife” include cost sharing policies, a departure from the standard full coverage provided by the German Social Health Insurance). None of the coefficients for these variables are significant, although including them in the model does not affect our basic findings. On the contrary: In this specification, the association of being in treatment group FULL with choosing lower levels of co-insurance is now significant in period 1 (p = 0,017) and in period 2 (p = 0,042).

Thus, the first and main finding of this research is:

Result 1: The data from our experiment provides evidence for *status quo* bias in health insurance policy choice.

Having established this for the first period of the experiment, we can now consider our second research question on the effect of experience on the *status quo* bias. For this purpose we investigate whether the difference in behavior observed between the groups in the first period carries over to repetitions of the task. In periods 2 and 3, subjects in group FULL still choose less and subjects in group SHARE more co-insurance than those in the BASELINE group (see Table 6). However, the differences are no longer statistically significant (p ≥ 0.302 for the comparison FULL/BASELINE; p ≥ 0.181 for SHARE/BASELINE; and p ≥ 0.214 for FULL/SHARE in Mann-Whitney U and Kolmogorov-Smirnov tests). Also, the average co-insurance choice per subject over all periods does not differ between the groups at the relevant levels of statistical confidence (p ≥ 0.142; see appendix for all p-values). Hence, our second result is

Result 2: The *status quo* bias diminishes in repeated decisions.

**Table 6:** Level of co-insurance (%) selected in periods 2–4, by group.

Period	Group	Mean	Median	SD
2	FULL	32.2	40	18.6
	BASELINE	35.8	50	20.2
	SHARE	37.8	50	16.8
3	FULL	27.4	30	20.9
	BASELINE	28.3	40	20.8
	SHARE	33.0	30	14.0
4	FULL	31.3	50	22.8
	BASELINE	27.1	30	20.3
	SHARE	32.6	40	18.9
All ^a^	FULL	28.5	32.5	14.4
	BASELINE	30.8	35	14.7
	SHARE	34.2	37.5	12.6

^a^ Mean of average co-insurance per subject across all four periods.

## 4. Conclusions

This paper reports on an economic experiment which examines whether decisions over health insurance policies are subject to *status quo* bias and, if so, whether experience mitigates this framing effect. A unique two-part design allows us to control for selection effects due to the subjects’ risk preferences. The results of our experiment indicate that *status quo* bias does play a role in consumer choices over health insurance policies. Subjects choose policies with different co-insurance arrangements depending on the particular *status quo* or default they face. However, the effect of the default framing does not hold up as subjects become more experienced in later periods of the experiment. Both results are consistent with previous research attesting to the presence of *status quo* bias in many settings as well as to the ameliorating effect of experience.

Several points may limit the applicability of our results in other contexts and require further investigation. For one, we find *status quo* bias among the group facing a full coverage *status quo* default, while the other treatment group with a maximum co-insurance *status quo* default made choices very similar to the control group. We presume this to be due to the coincidence of the optimal choice with the latter group’s framing, though a definitive answer would require further knowledge on the subjects’ utility functions. The same reasoning prevents us from making a final analysis of the effect of experience: While *status quo* bias disappears in our experiment as subjects gain experience, we cannot say whether behavior becomes more rational or merely shifts towards other non-rational patterns. There is literature to support both points (see above for the prior and [55] for an example of the latter). 

Nevertheless, the results of our experiment suggest implications for public policy on health insurance. Generally, it cannot be automatically assumed that prevailing choices on types of health insurance contracts reflect consumers’ preferences; they might rather mirror the circumstances of the decision environment. Full coverage insurance policies might be very common precisely because they are the default option. Inversely, this suggests that the decision environment can be modulated to favor welfare-enhancing market outcomes. If co-insurance were made the default option in public health insurance, *status quo* bias implies that a number of individuals would maintain this policy. A larger share of co-insurance contracts in the market could then serve to limit moral hazard and the demand for medical services and, in turn, alleviate some of the financial pressure on publicly financed health care systems. Of course, this laboratory experiment is best considered as the first link in a chain towards formulating health insurance policy. In a next step, these results and conclusions should be confirmed and studied on a larger scale and in a richer “real-life” decision environment, e.g., a field experiment.

As our second result suggests that *status quo* bias is an issue particularly among inexperienced consumers, any policy that capitalizes on this type of behavior is likely to be most successful among new customers first entering into the health insurance market. An alternative means of overcoming the drawbacks of *status quo* bias is to support consumers in becoming more experienced at choosing health insurance policies. If, for instance, periodic decisions over health insurance were implemented instead of letting policies roll over automatically, consumers would acquire experience and in the long run make choices more in line with their actual preferences.
